# Prevalence and predictors of renal dysfunction among people living with HIV on antiretroviral therapy in the Southern Highland of Tanzania: a hospital-based cross-sectional study

**DOI:** 10.11604/pamj.2022.41.137.27025

**Published:** 2022-02-17

**Authors:** Mololo Noah Mwanjala, Loveness John Urio, Majigo Venance Mtebe

**Affiliations:** 1 Muhimbili University of Health and Allied Sciences, Dar Es Salaam, Tanzania,; 2 Tanzania Field Epidemiology and Laboratory Training Program, Dar Es Salaam, Tanzania

**Keywords:** Antiretroviral therapy, glomerular filtration rate, HIV, renal dysfunction, Tanzania

## Abstract

**Introduction:**

infection with Human Immunodeficiency Virus (HIV) and use of antiretroviral therapy (ART) poses a significant risk of developing renal dysfunction in people living with HIV (PLHIV). Renal dysfunction contributes to the morbidity and mortality of PLHIV. There is limited information on renal dysfunction among PLHIV in the Southern Highland, the highest HIV prevalent area in Tanzania. We conducted a study to estimate the magnitude and predictors of renal dysfunction among PLHIV on ART.

**Methods:**

a cross-sectional study was conducted at Njombe Town Council Hospital from December 2019 to April 2020, recruiting 396 participants. Serum was obtained to measure creatinine level then calculated glomerular filtration rate (GFR) using CKD-EPI and the Bedside Schwartz equations. The participants' informations were collected using a structured questionnaire. Data analysis was performed using STATA version 15; a modified Poisson regression model was used to estimate prevalence ratios (PR). The level of significance was specified at 0.05.

**Results:**

the overall prevalence of renal dysfunction defined as GFR less than 90 mL/min/1.73 m^2^ was 20.7%, which increased by 4% as the age increases. The prevalence of renal dysfunction was higher in PLHIV on ART for more than six months to 24 months compared to their counterparts. Likewise, obese individuals had a 2.5 times higher prevalence of renal dysfunction than normal individuals.

**Conclusion:**

there is a relatively high prevalence of renal dysfunction among PLHIV on ART, predicted by age, duration on ART, and nutrition status.

## Introduction

Renal dysfunction is a common health problem worldwide, with epidemiological variations from one population to another [1]. Renal dysfunction is the kidney's impairment or abnormal functioning that leads to a decline of glomerular filtration. In Africa, 2% to 41% of people in the general population and 11% to 90% of people living with HIV (PLHIV) had renal dysfunction manifesting as Chronic Kidney Disease (CKD) in the year 2017 [2]. In Tanzania, studies reported a high prevalence of renal dysfunction among PLHIV in tertiary hospitals as 85.6% at Bugando Medical Centre, Northern Tanzania [3], and 32.8% at Muhimbili National Hospital, Eastern Tanzania [4].

Renal dysfunction contributes to significant morbidity and mortality for PLHIV [5]. Older people are at higher risk of developing renal dysfunction than the younger ones [6,7]. HIV replication in renal cells that damage the kidney and exposure to some antiretroviral drugs promotes renal dysfunction [8,9]. Additionally, the use of ART increases life expectancy but also is associated with increased risk of other non-communicable conditions such as diabetes mellitus and hypertension that contribute to renal dysfunctions [10].

Experience from clinical settings in Tanzania suggests infrequent routine monitoring of kidney status to PLHIV. Similarly, it is not common to calculate the glomerular filtration rate (GFR) in care and treatment clinics (CTC), leading to underreporting of renal dysfunction cases. There is insufficient scientific information on the magnitude of renal dysfunction in Southern Highland, the area with the highest prevalence of HIV in Tanzania [11]. The current study gives out the magnitude and factors associated with renal dysfunction among PLHIV on ART attending primary CTC in Njombe Region. The findings may help relevant health authorities to consider routine screening and subsequent renal disease treatment among PLHIV.

## Methods

**Study design and setting:** we conducted a hospital-based cross-sectional study design at Njombe Town Council Hospital in the Njombe Region, Southern Highland Zone of Tanzania, from December 2019 to April 2020. Njombe Region has a population estimate of 702,097 as per the 2012 census. The region lies between latitude 08°50' and 10°30' south of the equator and between longitude 33°45' and 35°45' east of Greenwich. Njombe had the highest prevalence of HIV in Tanzania, estimated at 11.4% for adults ≥15 years old and 2.3% for children under 15 years old by the year 2018 [11]. The CTC at Njombe Town Council Hospital serves almost 1800 active HIV clients every month.

**Sample size and sampling:** a minimum study sample size was estimated using Leslie and Kish formula; the calculation considered the prevalence of renal dysfunction among adult PLHIV starting ART in Mwanza, Tanzania [3]. A study enrolled a total of 356 PLHIV on ART. Self-reported and documented pregnant women were excluded because of a reported dynamic nature of GFR during the gestational period [12]. PLHIV were stratified based on duration on antiretroviral therapy use, as follows stratum 1: on ART for zero months up to six months, stratum 2: on ART for more than six months to 24 months, and stratum 3 - on ART above 24 months. Proportional to size sampling was used to determine the number of subjects to be selected from each stratum [13].

**Study variables:** the outcome variable was renal function status determined by GFR. The independent variables were age, sex, marital status, education level, occupation, residency, nutrition status, viral load count, CD4+ count, ART duration, HIV clinical stage, nutrition status, hypertension, and diabetes mellitus. Nutrition status was determined by Body Mass Index (BMI) ranges obtained by dividing weight in kilograms (kg) with squared height in meters (m) [weight (kg)/(height (m))^2^]. BMI was categorized as underweight (<18.5 kg/m^2^), normal weight (18.5 - 24.9 kg/m^2^), overweight (25.0 - 29.9 kg/m^2^), and obese (≥ 30 kg/m^2^) [14]. HIV clinal stage was classified as per WHO HIV clinical staging [15].

**Data collection:** information was collected using a structured questionnaire administered by trained CTC staff using the Swahili language (Tanzanian, native language). We collected socio-demographic information and clinical information from participants, and some extracted from participants' files. Weight in kilogram was measured using a standard weighing machine, while height in centimeter was measured using a height scale then converted into meters.

**Laboratory procedures:** venous blood from the arm's antecubital area was collected in a tube without anticoagulant by a trained phlebotomist. Blood was centrifuged within one hour at 1500 x g (relative centrifugal force) for 10 minutes at ambient temperature to obtain serum. The serum was placed into cryotubes and stored at -20°C until testing. All specimens were transported to the National Health Laboratory, Quality Assurance, and Training Center (NHL-QATC) at Dar Es Salaam for testing. Serum creatinine values were obtained using an automated chemistry analyzer, Cobas Integra 400 plus (Roche Diagnostic Ltd, Rotkreuz, Switzerland). The study used the NHL-QATC standard operating procedure for processing clinical chemistry samples. The quality control (normal and pathological) were performed before processing study samples and after every thirty samples.

**Estimation of glomerular filtration rate:** glomerular filtration rate (GFR) for participants aged 15 years and above was calculated using Chronic Kidney Disease Epidemiology Collaboration (CKD-EPI) equation:


$$GFR=\text{141 }\times {\text{ min(Scr/k,1)}}^{\text{a}}\times {\text{ max(Scr/k,1)}}^{\text{-1.209}}\times \text{0}{\text{.993}}^{\text{Age}}\times 1.018\left[if\text{ female}\right]\_1.159\left[if\text{ black}\right]$$

Where Scr is serum creatinine, κ is 0.7 for females and 0.9 for males, α is -0.329 for females and -0.411 for males, min indicates the minimum of Scr/κ or 1, and max indicates the maximum of Scr/κ or 1 [16]. CKD-EPI equation shows high performance and allows better renal function staging in HIV infected adults [17]. The Bedside Schwartz equation was used to calculate GFR for children less than 15 years old: GFR= 41.3 x (height/Scr), whereby Scr is serum creatinine in mg/dL and height in meters [16,18]. GFR was used to estimate each participant's renal function status; renal dysfunction was defined when GFR is less than 90 mL/min/1.73 m^2^.

**Data management and analysis:** the analysis was performed by using Stata version 15.1. The continuous variables were presented as mean (standard deviation) or median (interquartile ranges). Categorical variables were summarized in terms of frequencies and percentages. Chi-Square and Fisher exact tests were used to test the difference between proportions. The variables with a p-value of < 0.25 or considered necessary from the literature were retained in the regression model for controlling possible confounder. A modified Poisson regression model was employed to assess factors associated with renal dysfunction as an alternative to logistic regression because the prevalence was above 10% [19,20]. The strength of association was expressed using prevalence ratio (PR), defined as the prevalence of a disease among exposure over the prevalence of a disease among unexposed. Variables with p-value < 0.05 in multivariate analysis were considered statistically significant.

**Ethical issues:** the ethical approval (Ref. No.DA.287/298/01A) for the study was obtained from the Senate Research and Publication Committee, the Institutional Review Board of Muhimbili University of Health and Allied Sciences (MUHAS). Permission to conduct the study was obtained from Region Health Management and Hospital Management. Written informed consent was obtained from each participant/guardian before enrollment. The identification number was employed, and only authorized personnel had access to data.

## Results

**Socio-demographic characteristics of study participants:** a total of 396 PLHIV on ART were enrolled in this study. The majority of study participants (52.5%) were aged between 18 and 44 years, with a mean (± SD) of 41.5 (± 12.4) years. Most participants (59.6%) were female, and 71.5% were urban residents. More than half, 53.8%, were married or living with partners, 74.0% had primary education level, and 61.6% were farmers.

**Clinical characteristics of study participants:** Table 1 summarizes the clinical characteristics of study participants during ART initiation and at time study. During ART initiation: the majority of study participants (76.7%, n = 387) initiated a tenofovir (TDF) containing regimen. More than half of the participants (64.9%) had normal nutritional status based on BMI, and 210 (70.7%) participants had a CD4 count of ≤350 cells/mm^3^. Only 24.2% had HIV clinical stage IV during ART initiation.

**Table 1:** clinical characteristic of study participant during ART initiation and at time of the study

Variable	Frequency/mean/median	Percentage (%)
**Variable at ART initiation**		
ART regimen*		
TDF regimen	297	76.7
Non TDF regimen	90	23.3
**BMI**		
Underweight	51	12.9
Normal weight	257	64.9
Overweight	70	17.7
Obesity	18	4.5
CD4 count*		
≤ 350	210	70.7
>350	87	29.3
**HIV clinical stage**		
I	63	16.0
II	71	17.9
III	116	41.9
IV	96	24.2
**Variable at time of study**		
**ART duration (months)**		
Median (IQR)	68 (24.5, 112)	
0-6	35	8.8
>6-24	78	19.7
≥25	283	71.5
**ART regimen**		
TDF regimen	390	98.5
Non TDF regimen	6	1.5
Virological status*		
Viral load suppression	309	96.0
No viral load suppression	13	4.0
**BMI**		
Underweight	20	5.1
Normal weight	217	54.8
Overweight	103	26.0
Obesity	56	14.1
Diabetes mellitus	8	2.0
Hypertension	20	5.0

* Information for ART initiated regimen, CD4 count and viral load test was available for 387, 297 and 322 participants, respectively, out of 396 participants; TDF: tenofovir; BMI: body mass index; IQR: interquartile range

At time of the study, 71.5% of study participants were on treatment for more than 24 months, and 98.5% were taking TDF containing regimen. The median duration on ART was 68 months. Overall, 96.0% had viral load suppression defined as HIV viral load less than 1000 copies/mL. More than half (54.8%) of participants had normal BMI. A total of 8 (2.0%) individuals had known diabetes mellitus, and 20 (5.0%) had known hypertension (Table 1).

**Figure 1:**
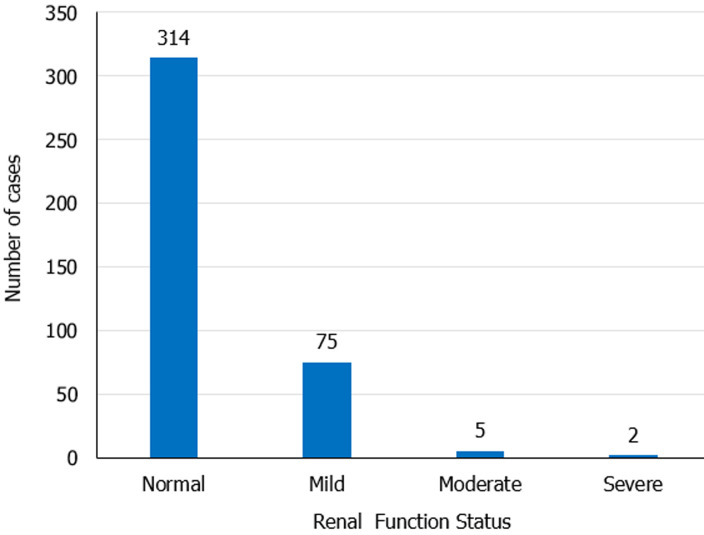
status of renal function among study participants

**Status of renal function:** of 396 study participants, 82 (20.7%) had decreased a glomerular filtration rate. Out of 82 participants with renal dysfunction, 75 (92%), 5 (6%), and 2 (2%) had mild, moderate, and severe renal dysfunction, respectively (Figure 1). There was no individual with kidney failure.

**Prevalence of renal dysfunction among PLHIV on ART:** the overall prevalence of renal dysfunction was (20.7%, 95%CI, 17.0 - 25.0). The renal dysfunction was significantly higher among participants aged 45 years old and above (28.2%) than the age group 18 - 44 years (14.9%), p=0.006. There were no significant differences in the proportion of renal dysfunction between participants based on sex, marital status, and place of residence (Table 2). A high prevalence of renal dysfunction was observed among participants on ART for more than six months to 24 months (38.7%) and obese (48.2%) than others in the same categories, p < 0.001. A high proportion of renal dysfunction was detected among participants with hypertension (35% vs 20%) and diabetes mellitus (25.0% vs 20.6%); however, the difference was not significant (Table 2).

**Table 2:** distribution of renal dysfunction by socio-demographic and clinical characteristics of study participants

Variable	Total	Renal dysfunction	Percentage (%)	95% CI	P-value
Overall renal dysfunction	396	82	20.7	17.0-25.0	
**Age (years)**					
<18	18	3	16.7	3.6-41.4	0.006
18-44	208	31	14.9	10.4-20.5	
≥45	170	48	28.2	21.6-35.6	
**Sex**					
Male	160	29	18.1	12.5-25.0	0.296
Female	236	53	22.5	17.3-28.3	
**Marital status**					
Single	135	24	17.8	11.7-25.2	0.064
Married/cohabiting	213	42	19.7	14.6-25.7	
Widowed/separated	48	16	33.3	20.4-48.4	
**Education level**					
Informal	49	11	22.5	11.8-36.6	0.510
Primary	293	63	21.5	16.9-26.7	
Secondary/tertiary	54	8	14.8	6.6-27.1	
**Occupation**					
Jobless	40	10	25.0	12.7-41.1	0.699
Farmer	244	51	20.9	16.0-26.5	
Employed	112	21	18.8	12.0-27.2	
**Place of residence**					
Rural	113	22	19.5	12.6-28.0	0.701
Urban	283	60	21.2	16.6-26.4	
**ART regimen**					
TDF regimen	390	82	21.0	17.1-25.4	0.207
Non TDF regimen	6	0	0	0-45.9	
**ART duration (months)**					
0-6	35	6	17.1	6.6-33.6	<0.001
7-24	78	31	39.7	28.8-51.5	
≥25	283	45	15.9	11.8-20.7	
Viral load suppression*	309	62	20.1	15.7-25.0	0.271
**Current BMI**					
Underweight	20	3	15.0	3.2-37.9	<0.001
Normal weight	217	37	17.1	12.3-22.7	
Overweight	103	15	14.6	0.8-22.9	
Obesity	56	27	48.2	34.7-62.0	
Diabetes mellitus	8	2	25.0	16.7-25.0	0.762
Hypertension	20	7	35.0	15.4-59.2	0.105

* The number of participants with viral load test was 322; BMI: body mass index; CI: confidence interval; TDF: tenofovir

**Predictors of renal dysfunction among PLHIV on ART:** in univariate analysis, age, marital status, duration on ART, and BMI associated with renal dysfunction. With an increase in age by one year, the prevalence of renal dysfunction increases by 3% (cPR, 1.03, 95% CI, 1.0-1.1, p < 0.001). Those on ART for more than six months to 24 months had 2 times the prevalence of renal dysfunction than those on ART for six months or less (cPR, 2.3, 95% CI, 1.1-5.0, p = 0.04). Obese participants have 2.8 times prevalence compared to those with normal weight (cPR, 2.8, 95% CI, 1.8-4.2, p < 0.001). Individuals who are not living with a life partner (divorced/widowed/separated) had a 1.8 times prevalence of renal dysfunction than those living single (Table 3).

**Table 3:** factors associated with renal dysfunction among PLHIV on ART

Variable	Total	Renal dysfunction	Univariate analysis		Multivariable analysis	
		N (%)	cPR (95%CI)	P-value	aPR (95%CI)	P-value
Age (years)	396	82 (20.7)	1.03 (1.0-1.1)	<0.001	1.04 (1.0-1.1)	<0.001
**Sex**						
Male	160	29 (18.1)	Ref			
Female	236	53 (22.5)	1.2 (0.8-1.8)	0.301		
**Marital status**						
Single	135	24 (17.8)	Ref			
Married	213	42 (19.7)	(0.7-1.7)	0.654	1.1 (0.7-1.8)	0.586
Widowed/separated	48	16 (33.3)	1.8 (1.1-3.2)	0.023	1.3 (0.7-2.4)	0.326
**ART duration (month)**						
0-6	35	6 (17.1)	Ref			
7-24	78	31 (39.7)	2.3 (1.1-5.0)	0.034	2.1 (1.0-4.5)	0.040
25+	283	45 (15.9)	0.9 (0.4-2.0)	0.850	0.8 (0.3-1.6)	0.578
**Virological status**						
VL suppression	309	62 (20.1)	2.6 (0.3-17.4)	0.322		
VL unsuppression	13	1 (7.7)	Ref			
**BMI**						
Normal weight	217	37 (17.1)	Ref			
Underweight	20	3 (15.0)	0.8 (0.2-2.6)	0.817	0.7 (0.2-2.5)	0.695
Overweight	103	15 (14.6)	0.8 (0.4-1.4)	0.576	0.8 (0.5-1.4)	0.570
Obesity	56	27 (48.2)	2.8 (1.8-4.2)	<0.001	2.5 (1.7-3.8)	<0.001
**Hypertension**						
Yes	20	7 (35.0)	1.7 (0.9-3.2)	0.081		
No	376	75 (20.0)	Ref			

VL: viral load; Ref: reference category predicted the probability of renal dysfunction; BMI: body mass index; cPR: crude prevalence ratio; aPR: adjusted prevalence ration

In multivariable analysis, age, duration on ART, and BMI were the only variable independently associated with renal dysfunction. One year increase in age had a 4% increase in prevalence of renal dysfunction (aPR, 1.04, 95%CI, 1.0-1.1, p < 0.001). Participants on ART for more than six months to 24 months had 2 times the prevalence of renal dysfunction than those on ART for six months or less (aPR, 2.1, 95%CI, 1.0-4.5, p = 0.04). Obese participants had 2.5 times prevalence than those with normal BMI (aPR, 2.5, 95%CI, 1.7-3.8, p < 0.001) (Table 3).

## Discussion

We found a considerable high prevalence by defining renal dysfunction as having GFR less than 90 mL/min/1.73 m^2^ (20.7%). Age, duration on ART, and BMI were independent predictors of renal dysfunction among PLHIV on ART. The current study's finding on the prevalence of renal dysfunction is comparable with a study conducted in Spain in which the overall prevalence was 25.0% [21] and in Ethiopia, where the prevalence was 25.4% [22]. However, some studies have reported a higher prevalence of renal dysfunction than our study's finding [8,23,24]. The studies conducted in tertiary health care settings reported 32.8% in Eastern Tanzania [4] and 85.6% in Northern Tanzania [3]. Differences in the study setting might explain the observed differences in the prevalence of renal dysfunction. Tertiary health care settings are likely to have individuals with significant renal dysfunction compared to primary health care settings where we conducted our study. We found an association between renal dysfunction and age; as age increases, the prevalence of renal dysfunction also increases. Similar findings have been reported in studies conducted in Tanzania [3,25] and from different parts of the world [6-8,23]. The study conducted in Mwanza, Northern Tanzania suggested that individuals who start ART at older age developed renal impairment at large as measured by the GFR [3]. The relationship of age and renal dysfunction calls for screening of renal function status to older clients before ART initiation and regular monitoring PLHIV on ART.

The duration of ART was associated with renal dysfunction. PLHIV on ART for more than six months to 2 years had more prevalence of renal dysfunctions than those on ART for up to six months. The same finding was reported in France [26] and agreed with different studies that documented the association between renal impairment and the use of ART [3,8,27]. Our finding is supported by Soto *et al*. clinical evidence, which testifies ART toxicity, resulting in a severe proximal tubular injury and proximal tubulopathy to HIV [28]. Additionally, nearly all study participants (98.5%) were on a TDF-containing regimen, which may have contributed to our findings on renal impairment, as described in other studies [10,26,29].

The other factor we found associated with renal dysfunction in the current study is being obese as defined by BMI of ≥ 30 kg/m^2^. Close to half of individuals with obesity were found to have renal dysfunction and had approximately 3 times the prevalence of renal dysfunction than those with normal BMI. Several studies reported a similar association of obesity with renal dysfunction results [3,21,30]. The biological explanation of the study finding is that most obese individuals have an increased risk of diabetes mellitus and hypertension, the traditional predictors of renal impairment [3,30].

The current study's major strength is the use of modified Poisson regression as an alternative method for classical logistic regression. The method provided the genuine depiction of estimated measures of effect while logistic regression tends to overestimate the effect mostly when the prevalence is more than ten [19,20]. This study's weakness is the failure to establish the causal and effect relationship due to the study design; however, it provides a snapshot of the burden of renal dysfunction.

## Conclusion

This study found a relatively high prevalence of renal dysfunction among PLHIV on ART predicted by age, duration on ART, and BMI. The findings suggest a need for routine screening and monitoring renal function status at CTC service delivery for early detection of kidney impairment for proper treatment.

**Funding:** this study received financial support from Tanzania Field Epidemiology and Laboratory Training Programme for data collection and laboratory work. The funder had no role in the study's design as well as the collection, analysis, interpretation of data, and writing of the manuscript.

### What is known about this topic

- Renal dysfunction is a devastating health problem in general population globally;
- Renal dysfunction is predicted by different factors such as age, use of ART, nutrition status and viral status.

### What this study adds

- Renal dysfunction is wide spread problem, can also found in PLHIV attending CTC in primary health care settings;
- Supporting the idea that there is possibility of recovering from renal dysfunction to PLHIV on ART for more than two years.
